# PhyloPen: Phylogenetic Tree Browsing Using a Pen and Touch Interface

**DOI:** 10.1371/currents.tol.d6d666469fc1942c665cb895b2305167

**Published:** 2015-11-23

**Authors:** Anthony Wehrer, Andrew Yee, Curtis Lisle, Charles Hughes

**Affiliations:** Department of Electrical Engineering and Computer Science, University of Central Florida, Orlando, Florida, USA; Department of Electrical Engineering and Computer Science, University of Central Florida, Orlando, Florida, USA; KnowledgeVis, LLC, Maitland, Florida, USA; Department of Electrical Engineering & Compter Science, Synthetic Reality Laboratory, University of Central Florida, Orlando, Florida, USA

## Abstract

Phylogenetic trees are used by researchers across multiple fields of study to display historical relationships between organisms or genes. Trees are used to examine the speciation process in evolutionary biology, to classify families of viruses in epidemiology, to demonstrate co-speciation in host and pathogen studies, and to explore genetic changes occurring during the disease process in cancer, among other applications. Due to their complexity and the amount of data they present in visual form, phylogenetic trees have generally been difficult to render for publication and challenging to directly interact with in digital form. To address these limitations, we developed PhyloPen, an experimental novel multi-touch and pen application that renders a phylogenetic tree and allows users to interactively navigate within the tree, examining nodes, branches, and auxiliary information, and annotate the tree for note-taking and collaboration. We present a discussion of the interactions implemented in PhyloPen and the results of a formative study that examines how the application was received after use by practicing biologists -- faculty members and graduate students in the discipline. These results are to be later used for a fully supported implementation of the software where the community will be welcomed to participate in its development.

## Introduction

Phylogenetic trees are a visual representation of the hierarchical relationships of common descent among a set of species or genes from a common ancestor. Often, these diagrams also include branch lengths to provide a time scale of the divergence of species or genes from their common ancestor. An example phylogenetic tree is shown in Figure 1.

Although there has been extensive work in the visualization and rendering of phylogenetic trees, relatively few systems have offered interaction with trees. Many existing tree rendering packages, such as FigTree, Mesquite, and others used by phylogenetics experts today, generate static renderings of tree diagrams (offering limited customization). Similarly, phylogenetic trees output from analysis programs (e.g. BEAST and Mr. Bayes) almost always have to be stylistically altered before presentation and/or publication, with branches colored and rotated, annotations added, and tip labels changed for readability. Without rendering flexibility, experts sometimes have to resort to manually adjusting the diagrams themselves using image editing software, such as Adobe Illustrator, which is a process that can be very time consuming. For these reasons, the goal of our work-in-progress application is to provide biology experts with an interactive experience that is efficient and hopefully more natural to use than existing tools.

We start by evaluating the impact and benefit of using pen and touch devices in browsing phylogenetic trees. It is for this purpose that we created an experimental novel pen and touch interface called PhyloPen to elicit formative feedback on this subject, as part of a larger NSF project called Arbor Workflows, a research effort to develop powerful, yet easy-to-use phylogenetic analysis tools for evolutionary biologists 1 ^,^ 2. In this paper, we discuss our preliminary work and findings on using pen and touch and provide a roadmap for our future development and user studies of the software on a full implementation scale. A unique contribution of this work is that it brings together the technologies of pen, touch, and annotation in a single system.

**An example phylogenetic tree of the Geospiza bird species from the Galapagos Islands rendered as a cladogram.**
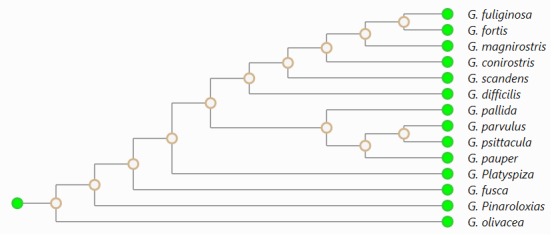

## Related Work

Although many of the commonly used phylogenetic tree viewers used by experts today generate only static renderings, OneZoom 3 is one exception that is dynamic and interactive although it does not explore non-traditional modes of input. OneZoom uses a fractal layout to handle trees, giving the user the option of changing the tree structure between different types of fractals and allowing the user to pan and zoom with a mouse through the tree to points of interest, refocusing its rendering on that area to provide more details about a particular region as the user zooms closer. The fractal layout of OneZoom gives it the additional benefit of being able to handle very large phylogenetic trees.

As for using different types of input from the traditional mouse and keyboard, there have been a number of previous efforts for the visualization of data using pen-, touch-, and other input-based interactive user interfaces, some of which were recently outlined by Isenberg and Isenberg 4 . There have been a few touch-based interfaces involving phylogenetic trees and species data specifically for museum displays, such as Involv 5 and the DeepTree exhibit 6 , as well as for collaborative learning, such as Phylo-Genie 7 . In regards to projects more closely aligning with PhyloPen’s goals, a pen and touch interface has also been implemented that is oriented toward phylogeny experts for co-located collaborative comparison tasks of different phylogenetic trees 8 . Although the majority of the focus was on touch-oriented features, the pen was especially useful for leaving sticky note annotations and integrated, free-floating annotations across the collaborative display. We are not aware of any other such interfaces that study the use of pen for phylogenetic tree browsing.

Unlike other interfaces, instead of using only touch for phylogenetic tree browsing, PhyloPen uses pen and touch together. Prior studies have shown potential benefit by using the two together. In a study by Brandl et al. 9 , the results of experiments between touch only, pen only, and pen and touch together suggested that the combination of pen and touch is superior in terms of speed and accuracy and is generally preferred by users. Additionally, in a study by Walny et al. 10 , the division of labor between pen and touch was also determined to be beneficial with touch being more natural to users for physical metaphor gestures (e.g., resizing, translating, etc.) and pen being better for gestures without corresponding physical actions in the real world or for writing (e.g., annotation) and drawing. The study also demonstrated that explicit mode switching can be minimized by using the two inputs together, potentially reducing the time to perform actions.

## Implementation

Our pen and touch user interface, PhyloPen, is written in C# using the Windows Presentation Foundation (WPF), which is a vector-based toolkit for creating user interfaces in Windows applications. WPF was chosen for its extensive and readily-available pen and touch API features. A full demonstration video is available online at the following address: https://www.youtube.com/watch?v=QEFqq4ZdLDc

**Graphical User Interface (GUI)**

The main components of our graphical user interface are the viewport to the left that contains the rendered phylogenetic tree and a resizable sidebar to the right, which contains various options and features related to the rendering. On the bottom, there is a status bar to specify the current context and status information regarding interactions with the tree rendering. The general layout of the user interface is illustrated in Figure 2. To operate on a specific phylogenetic tree, the tree is first loaded from an input file in standard format (e.g., Newick, phyloXML) into a NoSQL database (MongoDB). A list of the trees loaded into this database is then presented to the PhyloPen user for viewing and manipulation.

**The general layout of PhyloPen with the view of the phylogenetic tree to the left, an options sidebar to the right, and a status bar on the bottom.**
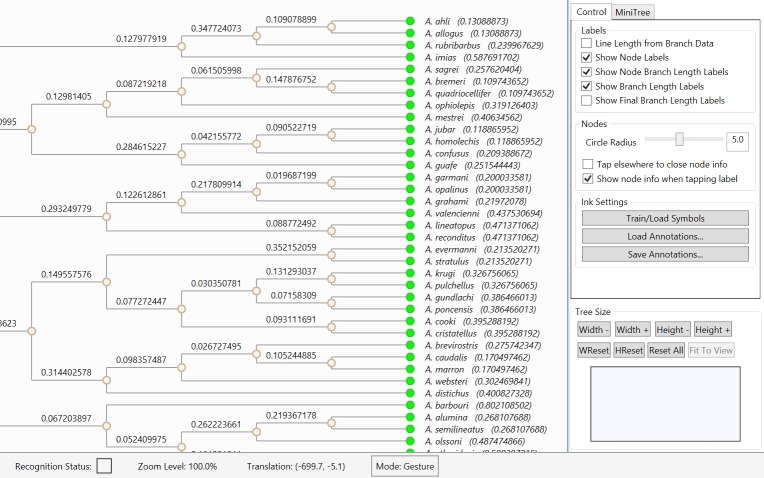

In the PhyloPen user interface, the main interaction features can be summarized into several categories: gesture-based navigation, annotation, layout modification, ancillary data display, and focus+context coordination. First, gesture-based navigation includes features that allow the user to translate the tree, zoom in and out of the view, and zoom to a particular region of the tree. Second, annotation includes features that allow the user to associate ink or text annotations to a clade in the tree and copy it to other clades in the tree. The third category, layout modification, consists of features where the layout or structure of the tree is altered by the user. These features include rotating nodes (e.g. swapping the order of child clades from a node), expanding and collapsing particular clades in the tree, and changing the width and height of the tree diagram. Fourth, ancillary data display includes features that involve information about the clades in the phylogenetic tree. Only minimal data have been placed into the tree rendering for the sake of experimentation and evaluation, but this feature category will be expanded upon in the future. Currently, when the user hovers over a leaf node with the pen, information is displayed about the species it represents, including the name of the species and an optional image of the species, the latter of which could benefit educational applications. Finally, the last category, focus+context coordination, contains features that are meant to help the user maintain context while interacting with the phylogenetic tree rendering. This category currently only contains a World-In-Miniature (WIM) model, a miniaturized version of the overall phylogenetic tree, which has a moving rectangular outline that shows where the user’s viewport is relative to the entire phylogenetic tree diagram. Additionally, when the user touches the WIM model with a stylus or finger, the view will move its center to the specific location that is touched. This feature is illustrated in Figure 3 in the tabbed sidebar of the user interface.

**The World-In-Miniature (WIM) tree can be used to find the user's current location in the tree as well as to jump to any location in the tree.**
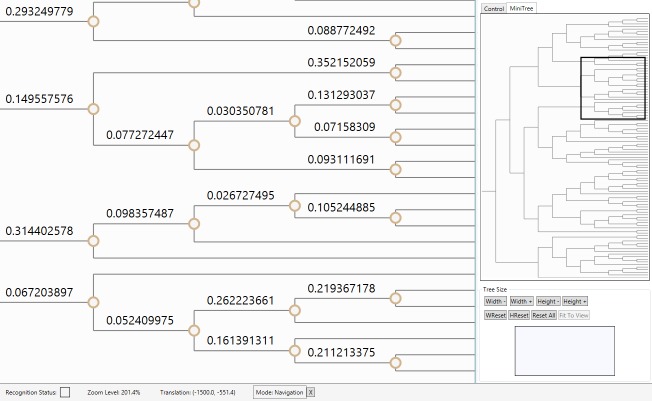

**Gestures**

Of all the features of PhyloPen, the gesture-based ones are the most important to our work. Our user interface implements gestures for two types of input: touch and pen. The touch-based gestures are primarily navigation-type, physical metaphor gestures while the ink gestures are primarily reserved for direct tree manipulation. For physical metaphor gestures, touch input tends to be more natural for people, as suggested by Walny et al. 10 . By separating the navigation gestures from the manipulation gestures, both can be used without explicit mode switching. However, for this version, we were initially asked by the biologists to allow for the possibility of performing ink gestures with their fingers, which is also useful for devices that do not have pens. This resulted in some explicit mode switching in our application between ink gesture mode and (touch) navigation mode. Measures were taken, however, to minimize its disruptiveness to the user, such as automatically switching back to the default ink gesture mode whenever the pen touches the screen and going into navigation mode automatically when two fingers are pressed on the screen at once. To exit navigation mode and return to the default ink gesture mode using only one’s fingers, the user can double-tap the screen as an alternative to a pen touch.

Since our application allows drawing through the touch interface, we have two different explicit modes with different sets of gestures, all of which are summarized in Table 1. In navigation mode, the user is able to translate and scale (zoom in and out of) the phylogenetic tree diagram using the typical touch gestures for these functions: swipe to translate and pinch to zoom. However, in ink gesture mode, there is a more general purpose set of gestures. This set currently includes gestures for the following actions: zoom to a region of the diagram, rotate children within a clade, expand and collapse clades, view information on leaf nodes, and annotate. To zoom to a certain region of the tree diagram, the user draws a rectangle around the desired area in one stroke. The tree will then gradually translate and zoom to that region, becoming the new viewport. To rotate a clade’s child nodes (e.g., reverse the clade’s child ordering), the user draws a C-like symbol over the branches of the clade to which it applies. This feature can be especially useful as a teaching tool to demonstrate the arbitrariness of clade ordering. To expand or collapse a node, the user draws a loop around the node. To temporarily display information on a node, the user must hover over the node. To keep this information visible, the user double-taps the desired node. Also, to start annotating a node, the user simply draws an X symbol anywhere on the ink canvas.

**An example of the interface demonstrating the process of creating an annotation. The above annotation was just written and has yet to be assigned to a node.**
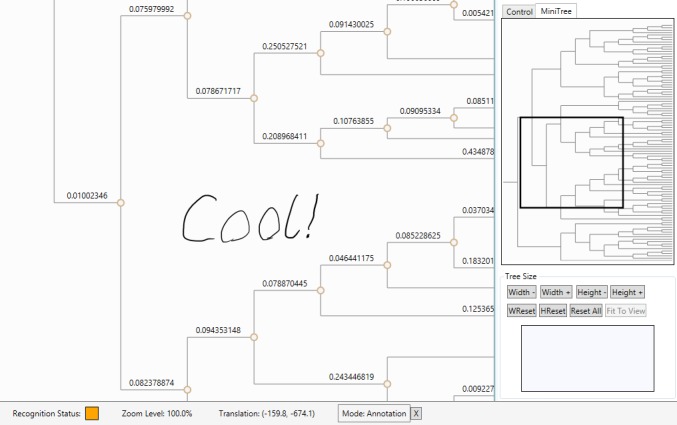

Once the user signals the start of an annotation, the interface enters annotation mode, a distinct third mode. The user may write annotations using ink anywhere on the ink canvas (as shown in Figure 4) and then double-tap a node to associate with the annotation. Upon associating the ink annotation, a small color-coded annotation square appears next to the node (in the place of the node circle), which displays the annotation upon hover. Each node can have multiple annotations. Each new one is partially stacked over the older ones. An annotation can be held open, just as with node information, by double-tapping the square instead of hovering. If there are multiple annotations, a special annotation selection menu appears to give the user a choice between each color-coded box to eliminate the struggle of picking the correct entry by a small margin of tapping error. This selection menu, as well as the color-coded annotation squares, are illustrated by Figure 5. Another implemented feature, requested by our test participants, allows an annotation assigned to a specific clade to be copied to its ancestors or descendants by swiping left or right, respectively, from within the annotation square.

**When there are multiple annotations in the stack for a specific clade and the user wants to display one, a special menu appears to make it easier for the user to select the desired annotation.**
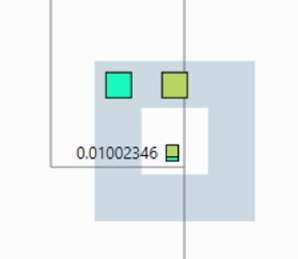

**Figure:**
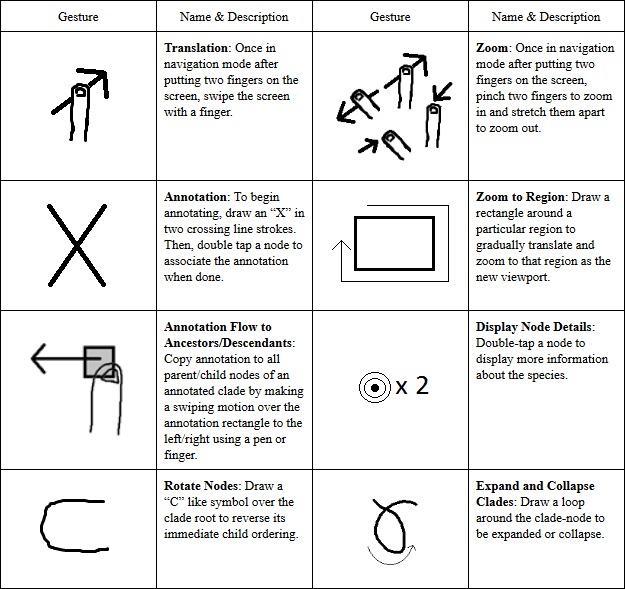
Table 1: PhyloPen has a number of different touch- and ink-based gestures to help browse the phylogenetic tree diagram

Besides the gestures listed in Table 1, there is one additional gesture-based feature in PhyloPen. On the user interface sidebar, there are buttons for changing the width and height of the tree by a fixed amount. However, we also include a gesture-based region for resizing the tree layout, which we refer to as the tree resize canvas. It is shown as the small rectangular ink canvas in the bottom-right corner of the GUI in Figure 2. When a line is drawn on the miniature canvas, the change in width and height of the tree depends on the direction, angle, and length of the stroke. This is illustrated in Figure 6.

**The tree resize ink canvas can change the width and height of the tree.**
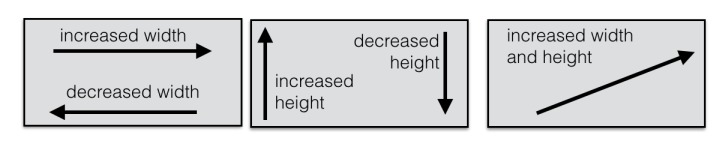

## User Evaluations

We conducted a formative user evaluation of PhyloPen, involving three Ph.D. students and three faculty members from one of our biological collaborators’ institutions, to gauge the interface’s usefulness and usability. Each participant individually tested PhyloPen on a Microsoft Surface Pro 2 with pen and touch support and responded to a post-questionnaire. In the post-questionnaire, users were asked to rate each feature of the user interface on a 5-point Likert scaling according to its usefulness and usability, separately. In terms of usefulness, the ratings were labeled as hardly useful, a little useful, pretty useful, really useful, and extremely useful. The usability was expressed in similar terms (labeled as “fun”). Quantifying these ratings from 1 to 5, the average rating for each feature is shown in the bar chart in Figure 7. The users were also given a variety of other questions in the post-questionnaire to provide feedback on how to direct future development.

**The bar chart shows the average Likert ratings from users on features of PhyloPen in terms of usefulness and perceived usability.**
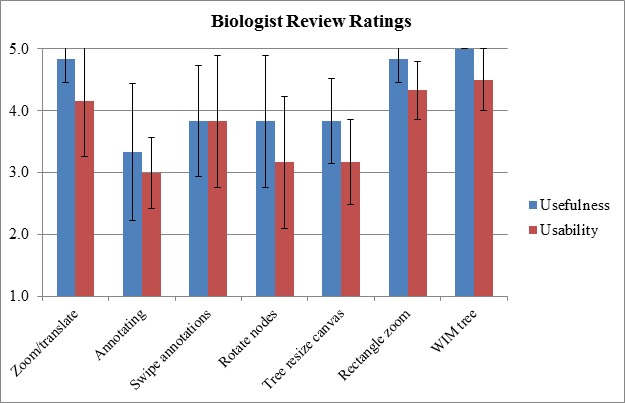

For the most part, according to Figure 7, the relative usefulness of a feature, as perceived by reviewers, seems correlated with the usability of the feature. Annotation has the lowest score in terms of usefulness, and one reason may be that annotations can only be stored as strokes, not recognized text. Also, many users seemed to experience some degree of frustration with the system. One potential source of frustration for the users may be having to double-tap a tiny node circle with the pen in order to associate the annotation. Although it is possible to enlarge the circles, we feel it may prove better to implement a circular gesture to surround the node for selection. This should be less concentration-intensive for the user. There are also cases where the user may want to delete the annotation from certain nodes after passing it up or down the tree (e.g., annotating the bird clades with "can fly" and then removing it from the clades of ostriches, rheas, kiwis, etc). We are not certain how best to implement a group annotation deletion gesture. Due to these limitations, we feel the annotation system may need to be revamped to handle a more flexible approach that is more comfortable for the user.

Two other features that scored relatively low in terms of usability were node rotation and the tree resize canvas. Currently, the two children flip instantaneously after the rotate gesture is performed. However, when this flip is performed over a large area of the tree diagram, an instantaneous change results in context loss and potential confusion of the user. We hypothesize that there would be higher scores for rotating clade children if we animated the rotation process. As for the tree resize canvas, we discovered that there was some confusion between the coordinate system of the tree resize feature and the coordinate system of the main canvas. Our tree resize canvas feature uses a standard Euclidean coordinate system, but the graphical components in the main canvas are stored in a coordinate system with an inverted y-axis. Thus, when the tree resizes in height, the tree grows downward. However, we have the users drawing up in the direction opposite of growth, making the feature far less intuitive than if the tree grew in the direction the user draws.

One perplexing result was that the translate/zoom feature was rated higher in usefulness than in usability. However, we believe that this could be the result of issues we experienced with touch features using WPF where the transformations were not as smooth as would be expected or the touch input itself would occasionally stop being received by the program with seemingly no explanation.

Overall, the feedback from the biologists was positive despite a few points of frustration. The users were generally able to figure out how to use the interface after watching a short demonstration (although one individual expressed the desire for assistance with remembering the gestures, such as a short list/cheat sheet of all the ink gestures). The biologists found the touch navigation relatively natural and intuitive while other features still needed improvement, as noted earlier. Additional features were also suggested, the most common of which was to move a clade within a tree by re-attaching it to an alternative ancestor.

## Moving Forward

From the start, we worked on this version of PhyloPen as an experimental build. As such, we did not necessarily intend to make it available to the general public and to support it. However, this paper serves as an invitation to the biological community to work with us, giving feedback in creating the next generation of PhyloPen, which will be made available and open source. In this way, we will not be working in isolation, separated from the community that we wish to serve, because it is the community that best knows what it wants and needs. The new version will account for the feedback given by the users already contributing to the evaluation and will be open to more changes based on what the community finds most appropriate. When ready, a new version of PhyloPen will be released on the Arbor Workflows GitHub (https://github.com/arborworkflows). For each version released, the users will be able to test the software and fill out a post-questionnaire, offering feedback on their overall experience, how to improve what is available, and what features are highest in priority that have not already been implemented. The software resulting from this development process will ultimately be given a summative evaluation with a larger group of people than the experimental version.

## Conclusions

Our objective is to develop an interactive phylogenetic tree-browsing user interface for experts that is more dynamic and ideally more natural than commonly used browsers, ultimately allowing the user to navigate, annotate, and change the tree structure with ease. In this work-in-progress, we tested an experimental pen- and touch-based interactive browser we developed called PhyloPen. Our preliminary formative evaluations show positive results, demonstrating some usefulness of pen and touch interfaces for phylogenetic trees. However, additional work based on our formative evaluations, including the revision of existing features and the addition of new ones (e.g., cut and reattachment for clades), and a thorough summative evaluation will be needed to draw more definitive conclusions. In support of this quest, we invite the biological community to work alongside us in reaching that goal, giving their opinions and shaping what PhyloPen ultimately should be.

## Competing Interests

The authors have declared that no competing interests exist.
